# Topical Microemulsions: Skin Irritation Potential and Anti-Inflammatory Effects of Herbal Substances

**DOI:** 10.3390/ph16070999

**Published:** 2023-07-13

**Authors:** Jiraporn Leanpolchareanchai, Veerawat Teeranachaideekul

**Affiliations:** Department of Pharmacy, Faculty of Pharmacy, Mahidol University, Bangkok 10400, Thailand; jiraporn.lea@mahidol.ac.th

**Keywords:** anti-inflammatory, herbal substances, microemulsions, skin irritation, topical application

## Abstract

Microemulsions (MEs) have gained prominence as effective drug delivery systems owing to their optical transparency, low viscosity, and thermodynamic stability. MEs, when stabilized with surfactants and/or co-surfactants, exhibit enhanced drug solubilization, prolonged shelf life, and simple preparation methods. This review examines the various types of MEs, explores different preparation techniques, and investigates characterization approaches. Plant extracts and bioactive compounds are well established for their utilization as active ingredients in the pharmaceutical and cosmetic industries. Being derived from natural sources, they serve as preferable alternatives to synthetic chemicals. Furthermore, they have demonstrated a wide range of therapeutic effects, including anti-inflammatory, antimicrobial, and antioxidant activities. However, the topical application of plant extracts and bioactive compounds has certain limitations, such as low skin absorption and stability. To overcome these challenges, the utilization of MEs enables enhanced skin absorption, thereby making them a valuable mode of administration. However, considering the significant surfactant content in MEs, this review evaluates the potential skin irritation caused by MEs containing herbal substances. Additionally, the review explores the topical application of MEs specifically for herbal substances, with an emphasis on their anti-inflammatory properties.

## 1. Introduction

**Figure 1:**
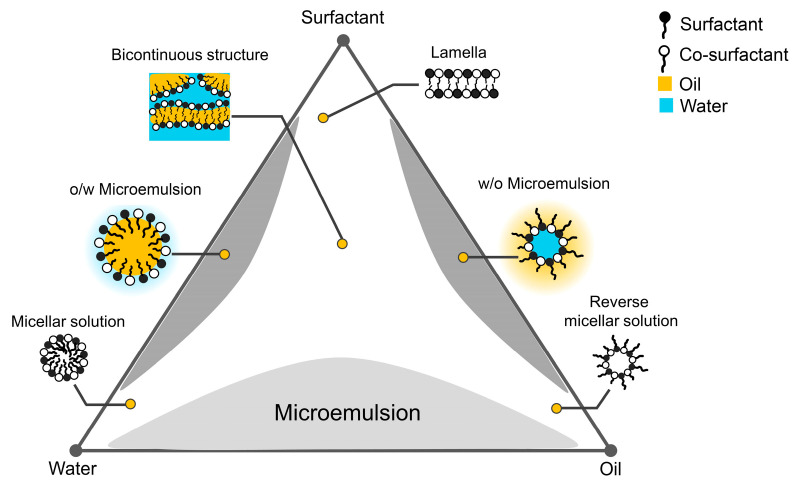
The ternary phase diagram depicts various structures.

The discovery of microemulsions (MEs), an intriguing delivery system, dates back to 1943 when Hoar and Schulman first recognized their potential. Hoar and Schulman created an isotropic and non-conducting solution by mixing a milky solution containing long-chain fatty acids with medium- or short-chain alcohols [1]. Due to a lack of established terminology at that time, these structures were referred to as “soluble oil” and “oleopathic hydro-micelle” [2,3,4]. In 1981, Danielsson and Lindman first described MEs as a system composed of water, oil, and amphiphiles that formed a single optically isotropic and thermodynamically stable liquid solution [5]. A mixture of oil, water, surfactant, and co-surfactant can form various association structures, including MEs, depending on the chemical nature and concentration of the elements [6,7]. The association structures, as illustrated in Figure 1, include MEs and regular emulsions (e.g., oil-in-water (o/w) and water-in-oil (w/o)), as well as micellar and mesomorphic phases with various arrangements such as lamellar, hexagonal, cubic, and various gels, and oily dispersions [6,8].

**Figure 2:**
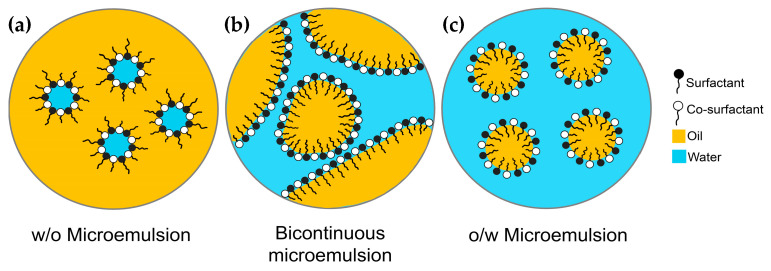
Schematic images of MEs types: (**a**) w/o MEs, (**b**) bicontinuous MEs, and (**c**) o/w MEs.

The ternary phase diagram (Figure 1) illustrates the equilibrium of various phases based on all the possible combinations of the three components at a constant temperature and pressure. Constructing a ternary phase diagram is a valuable technique for elucidating the sophisticated series of interactions that occur when different ratios of components are mixed. MEs and other associated structures are part of the ternary phase diagram. MEs are thermodynamically stable, isotropic mixtures of water and oil stabilized by surfactants and/or co-surfactant molecules [9,10,11]. The droplet sizes of the MEs are less than 100 nm [12,13,14]. When oil, water, and surfactants are combined in appropriate proportions, MEs are formed as a single structure. Their structures are influenced by a variety of factors, including the ratio of their constituents, the chemical composition of the substances involved, as well as temperature and pressure [6,15]. As depicted in Figure 2, the three primary types of MEs are o/w, bicontinuous, and w/o [16,17,18].

MEs are extensively used in the pharmaceutical and cosmetic industries as a type of emulsion technology [9,19,20]. They offer several advantages, including improved drug solubility, increased bioavailability of poorly water-soluble drugs, and enhanced chemical stability [16,21,22,23]. The intensive utilization of MEs spans various administration routes, including oral [24,25,26,27], topical [28,29,30,31], and parenteral [32,33,34,35], establishing them as promising carrier systems for delivering a broad spectrum of active ingredients, including drugs that are either water-soluble or poorly water-soluble [11,36].

MEs have been the subject of extensive research as delivery systems for topical and dermal applications, particularly because of their ability to enhance skin penetration [36]. They have been reported to increase the thermodynamic activity of drugs intended for topical use, resulting in improved drug absorption through enhanced diffusion into the skin. This property has garnered significant interest from researchers investigating drug delivery systems for topical and dermal applications. Moreover, they contain high concentrations of surfactants and/or co-surfactants that act as enhancers. These enhancers have been shown to reduce the barrier function of the stratum corneum, allowing increased drug penetration into the skin. The distinct combination of enhanced drug activity and reduced barrier function has rendered MEs highly attractive to researchers for developing drug delivery systems for topical and dermal applications [21,36]. Furthermore, the water and oil components of MEs have been found to improve skin hydration and occlusive effects, thereby enhancing drug penetration through the skin [36].

In general, two methods are used for preparing MEs: spontaneous emulsification and phase inversion technique (PIT) [37]. Spontaneous emulsification involves the construction of a pseudo-ternary phase diagram by systematically mixing the oil phase with surfactants and/or co-surfactants in various ratios. Water is then added incrementally, and the samples are visually observed for the presence of monophasic or biphasic systems. A phase diagram is plotted, and the ME region is identified [38]. In contrast, the PIT method is used to prepare MEs by changing the temperature, which causes a phase inversion [38].

Owing to the complexity, variety of structures, and various components of ME system, several techniques are usually required to obtain more information about the structure of MEs. Characterization of MEs includes microscopic techniques, viscosity, conductivity, differential scanning calorimetry (DSC), spectroscopic techniques, and particle size analysis by dynamic light scattering (DLS) [39]. The following are brief details of each technique:

- Microscopy techniques can be used to identify ME types (o/w and w/o) using water-soluble (e.g., brilliant blue) or oil-soluble (e.g., Sudan IV) dyes [40,41,42].
- MEs can be distinguished from liquid crystals using cross-polarized light microscopy. Unlike lamellar and hexagonal liquid crystals, which are anisotropic substances, MEs do not exhibit birefringence under cross-polarized light due to their isotropic properties [40,43,44,45,46].
- Conductivity measurement serves as a means to differentiate ME types. The o/w MEs, with water as the external phase, exhibit conductivity, whereas the w/o MEs, with oil as the external phase, act as insulators. This technique is useful for investigating ME structure and phase transitions with increasing water content [47,48].
- Electron microscopy techniques such as transmission electron microscopy (TEM) and scanning electron microscopy (SEM) are commonly used to assess ME morphology [49,50,51,52], providing higher resolution than optical microscopes [39,53]. TEM images are two-dimensional, generated by transmitting electrons through the sample. These images often require staining with heavy metal compounds, such as uranyl acetate or phosphotungstic acid, as contrast agents and the samples are dried before measurement [54,55,56]. SEM is employed to directly visualize the surface features of samples by generating three-dimensional images through the detection of backscattered and secondary electrons [39,53]. Cryo- and freeze-fracture techniques have been developed to minimize the drying artifacts when investigating ME structures using these methods [52,57,58,59,60,61].
- Typically, MEs exhibit low viscosities and exhibit Newtonian flow behaviors [56]. However, some studies have reported non-Newtonian behaviors such as shear thinning [47,62,63,64].
- DLS is employed to analyze droplet size and polydispersity index (PDI) of MEs by detecting scattered light intensity as particles undergo Brownian motion. The hydrodynamic radius was calculated using the Stokes-Einstein equation [65]. PDI values range from 0 to 1, with values below 0.1 indicating high monodispersity, 0.1 to 0.4 indicating moderate polydispersity, and values above 0.4 indicating high polydispersity [65,66].
- Zeta potential (ZP) is utilized to predict colloidal dispersion stability, with its value dependent on pH, ionic strength, and particle concentration. ZP measurement is common for o/w MEs; however, some studies have reported ZP values for w/o MEs [54,59,67,68,69]. ZP values greater than ±30 mV indicate excellent stability [65]. Other factors, such as steric hindrance, also contribute to the stability of colloidal dispersions stabilized by nonionic surfactants [70].
- DSC is employed to characterize MEs and differentiate between “bulk” and “bound” water [40,71,72]. Water in o/w MEs exhibits properties similar to those of pure water, while bound water in w/o MEs exhibits different characteristics [40,43], resulting in a lower freezing peak of water in w/o MEs compared to o/w MEs [73,74]. However, the addition of co-solvents such as pentylene glycol or propylene glycol (PG) may affect the freezing peak in the DSC thermogram [73,74].

## 2. Human Skin Structure

**Figure 3:**
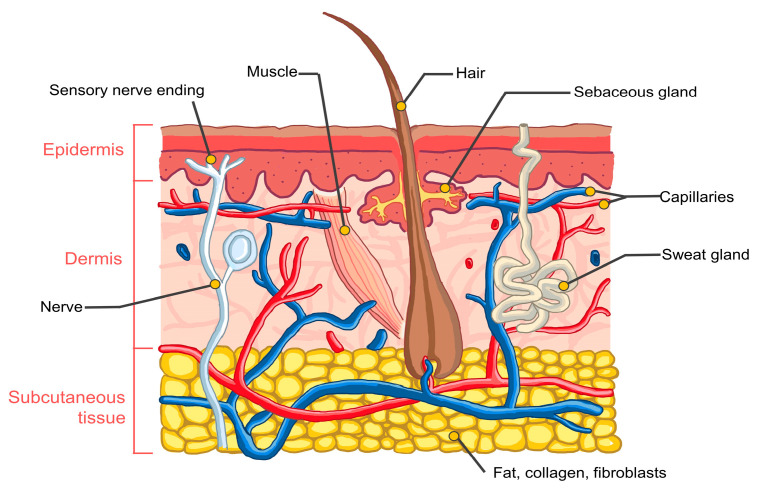
Schematic images of human skin.

The skin is the largest organ of the body and is a complex organ containing many types of cells. The skin structure is illustrated in Figure 3.

The skin accounts for 12–16% of an adult’s body weight and consists of three main layers: the epidermis, dermis, and subcutaneous tissue [75,76]. The subcutaneous layer serves as a bridge between the dermis and underlying tissues, providing insulation and protection against impact. The dermis is composed of connective tissue, collagen fibers, and elastic tissue and provides skin elasticity. Components found in the dermis include blood vessels, lymph vessels, nerve endings, sweat glands, sebaceous glands, and hair follicles.

The outermost layer of the skin, the epidermis, acts as a barrier against external factors and varies in thickness (0.06–0.80 mm). It comprises four sublayers: stratum corneum, stratum granulosum, stratum spinosum, and stratum basale. The stratum corneum, the outermost sublayer, consists of 20–30 layers of flattened corneocytes surrounded by a lipid bilayer [77]. This prevents unwanted substances from entering and prevents water loss from the body [78]. The stratum granulosum beneath the stratum corneum contains keratohyalin and lamellar granules that hold cells together. The stratum spinosum, also called the prickle cell layer, contains keratinocytes held together by desmosomes [79] and Langerhans cells that are responsible for immune reactions [80]. The basal layer, or the stratum germinativum, connects to the dermis and contains melanocytes and Merkel cells, which are involved in melanin production and light-touch sensation, respectively [81].

## 3. Skin Irritation and Mechanism

Skin irritation occurs because of unspecified damage from contact with external stimuli or agents, increasing sensitivity and causing inflammatory reactions. It has a unique pathophysiology, natural history, and clinical manifestations and presents in various ways depending on factors such as the environment, host, and stimuli. Skin irritation refers to the reversible skin damage caused by a test chemical applied for up to 4 h, whereas skin corrosion involves irreversible damage, necrosis, ulcers, bleeding, and bloody scabs. The main pathogenic mechanisms of skin irritation include skin barrier damage, cytokine cascade production, and oxidative stress, leading to visible and subclinical inflammatory reactions. Necrosis and ulceration are the common causes of corrosion [82,83].

Distinguishing between irritation contact dermatitis (ICD) and allergic contact dermatitis (ACD) based on clinical signs alone is challenging, as both involve cytokines and intercellular interactions [84]. ICD is characterized by stratum corneum rupture, epidermal necrosis, and hyperproliferation, whereas ACD typically presents with vesicle production.

In ICD, irritants penetrate the epidermal barrier, damage keratinocytes, and cause nonspecific T-cell activation. Epidermal keratinocyte injury initiates an inflammatory process leading to ICD. When irritants harm the stratum corneum, keratinocytes release interleukin (IL)-1α, which stimulates the production of IL-1α, IL-1β, IL-6, IL-8, and tumor necrosis factor (TNF)-α from surrounding keratinocytes and fibroblasts, resulting in skin irritation and epidermal inflammation [85,86].

Unlike ICD, ACD involves the activation of hapten-specific T-lymphocytes, leading to distinct molecular expression profiles [85,87,88]. Chemokine genes, including CXCL9 and CXCL10, which are regulated by T-cell effector cytokines, exhibit different levels of upregulation in hapten-specific skin inflammation but are not induced during irritant-induced skin inflammation [85].

Surfactants are commonly used in cosmetics, such as shower gels, hair conditioners, skincare products, and facial washes. They can induce irritant reactions owing to their ability to solubilize skin lipid membranes. Surfactant molecules contain both polar and nonpolar parts, with the nonpolar portion predominantly composed of flexible hydrocarbon chains. Surfactants are categorized into four main types based on their hydrophilic components: anionic, cationic, nonionic, and amphoteric surfactants [89].

Surfactants have the potential to induce skin irritation depending on their physicochemical properties [90,91]. Anionic surfactants find widespread usage in different products, frequently leading to irritation to humans and animals. Cationic surfactants are as irritating as anionic ones due to their cytotoxicity. Nonionic surfactants, frequently utilized in skincare products, have a lower likelihood of causing irritation [91]. They are also formulated in combination with anionic and cationic surfactants to reduce the risk of skin irritation. Amphoteric surfactants containing both anionic and cationic groups are pH sensitive, and their properties are significantly influenced by pH. They exhibit anionic characteristics at high pH values and cationic characteristics at low pH values.

**Figure 4:**
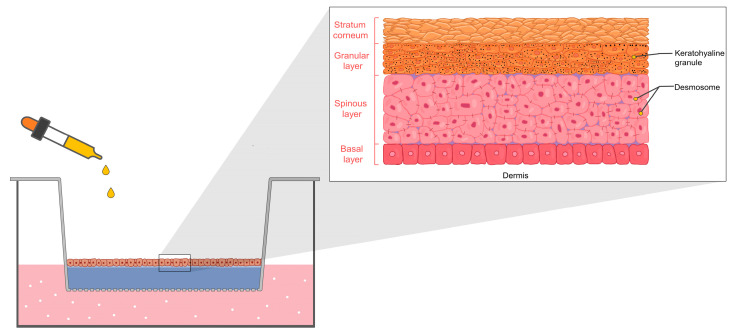
In vitro skin irritation of the RhE model in transwell.

The Draize test, described in the Organisation for Economic Co-operation and Development (OECD) 404, uses animals, preferably albino rabbits, to assess skin irritation by administering a single dose of a test chemical for 4 h. Clinical observations and skin reaction grading for erythema and edema are performed at various time points after patch removal [92]. The observations continued for 14 days to distinguish between skin irritation and corrosion. However, this test has been criticized for its subjectivity, overestimation of human responses, and cruelty [93]. In 2013, the European Union banned animal testing of cosmetics, leading to alternative in vitro methods using reconstructed human epidermis (RhE) (Figure 4) [94,95]. This alternative, approved by the OECD (test number 439), closely mimics the human epidermis with a multi-layered differentiated structure resembling human skin layers [96].

To evaluate skin irritation in RhE, cell viability is measured using enzyme conversion of vital dyes (e.g., 3-(4,5-dimethylthiazol-2-yl)-2,5-diphenyl-2H-tetrazolium bromide (MTT) and thiazolyl blue) and compared to untreated controls. Test substances with cell viability ≤50% are considered irritants. Seven commercially approved RhE skin models, including EpiSkin^TM^, EpiDerm^TM^ SIT, SkinEthic^TM^ RHE, LabCyte EPI-MODEL 24 SIT, epiCS^®^, Skin+^®^, and KeraSkin^TM^ SIT, are officially included in OECD 439 [96].

## 4. Skin Irritation Potential of MEs Containing Herbal Extracts

Herbal extracts and their active components have found widespread use in topical applications. However, a limitation of this method is the suboptimal absorption of these substances into the skin. Consequently, MEs have been extensively used to improve the absorption of herbal compounds, thereby enhancing their therapeutic efficacy. MEs, with their high surfactant concentrations, can facilitate the skin absorption of encapsulated drugs; however, they may also lead to skin irritation. However, studies have suggested that MEs are safe and do not irritate the skin [97,98]. Table 1 shows studies exploring the skin irritation potential of MEs containing herbal substances. For instance, Vibhute et al. developed a *Moringa oleifera* seed oil ME formulation for topical anti-inflammatory treatment using Tween^®^ 80 (10.03%), Span^®^ 80 (48.99%), and water (15.68%). In the three albino rabbits tested, the ME formulation did not cause erythema, edema, or inflammation after 72 h, indicating its non-irritating and non-sensitizing properties on the skin [97].

Guo et al. [98] developed salvianolic acid B-loaded ME using a surfactant and a co-surfactant (polyethylene glycol (PEG)-40 castor oil:Tween^®^ 80:PEG 400:1,2-PG, 10:1:1:1). They tested the skin-irritation effects of this ME formulation in an imiquimod-induced mouse model of psoriasis-like dermatitis. The ME treatment group exhibited a similar pathology to that of the control group but with a lower severity index. This suggested that the salvianolic acid B-loaded ME did not cause skin irritation.

Marsup et al. [99] created a topical ME serum with 1% *Cordyceps militaris* extract stabilized with Tween^®^ 85 (26.67%) as a surfactant and tested skin irritation in 30 healthy volunteers. When applied under occlusive conditions for 4 h, skin irritation was assessed as redness, edema, and dryness after 24, 48, and 72 h. No signs of irritation were observed in this study.

Parveen et al. [100] developed a topical o/w ME with pomegranate extract, using Tween^®^ 80 as a surfactant. They tested the potential for skin irritation on volunteers’ forearms over a 48-h period. The results revealed that this formulation did not cause skin irritation.

Lin et al. [101] formulated 2.6% catechin-loaded ME containing a mixture of Brij 30 and Brij 35 (20%) for topical use. The potential of this ME formulation to cause skin damage was assessed in rats by observing histopathological changes after 24 h of application. The results revealed a lack of significant irritation, indicating that the ME exhibited satisfactory skin compatibility.

Puri et al. [102] developed topical o/w ME with 0.2% dibenzoylmethane (DBM) intended for treating ultraviolet (UV)-induced photoaging. The formulation was prepared using Tween^®^ 80 (32%) and n-butanol (16%). Skin sensitivity and histopathological studies were performed on mice by evaluating the skin redness and histological changes after 4 h of application. The results revealed normal skin sections without any pathological alterations, confirming the safety of DBM-loaded ME.

Gandhi et al. [103] studied the skin irritation associated with a ME gel loaded with clove oil, which consisted of 7.8% of a mixture of Tween^®^ 80. The gel was applied to rat skin for 24 h, and no irritation was observed compared to the control group, indicating that ME gel with clove oil was safe for topical use.

Brathwaite et al. [104] developed ME loaded with *Pouteria macrophylla* fruit extract for skin depigmentation. The formulation consisted of 40% Cremophor^®^ ELP and Span^®^ 80 in a 4:1 ratio, with 10% ethyl oleate as a co-surfactant. The formulation was tested for skin irritation potential using EpiSkin™. The formulation was established to be non-irritating, with 75.54% cell viability. This was higher than the cutoff value of over 50%, which typically indicates skin irritation.

From the above findings, we can deduce that despite having a high surfactant concentration, MEs do not cause skin irritation, which renders them suitable for topical applications. Furthermore, studies have demonstrated the potential of plant-based ME-containing extracts for the treatment or minimization of skin irritation. For example, d-limonene, the main oil constituent of citrus fruit peel, is a known skin irritant and sensitizer [105,106]. MEs containing d-limonene were developed and tested on mice over seven consecutive days to evaluate their skin irritation potential [105]. These ME formulations contained 12.50% d-limonene, 12.50% Gelucire^®^ 44/12, 46.64% Labrasol^®^, and 11.66% Labrafil^®^ M 1944. The results indicated that d-limonene MEs caused significantly reduced irritation compared to pure limonene. Additionally, the histological scores revealed that the ME formulation was potentially less damaging. The authors concluded that encapsulation of limonene oil in MEs may offer protection against the irritant effects of pure limonene oil. Another example is a citrus essential oil-loaded ME formulation [106]. Limonene, the main component of citrus essential oils, causes skin sensitivity upon prolonged exposure to air. To mitigate the skin-irritation potential of limonene, the citrus essential oil was encapsulated in a ME containing 5.0% essential oil, 26.7% decyl glucoside, 13.3% butylene glycol, and 55.0% water. Skin irritation tests were conducted on 30 participants, and the results indicated that the application of this ME formulation caused significantly reduced irritation compared to the application of pure oil. This implies that MEs are safer alternatives for topical applications.

Moreover, it was reported that MEs containing jojoba oil as the oil phase reduced the potential skin irritation caused by irritating drugs. For example, tazarotene is a synthetic retinoid that functions as a topical antiproliferative and anti-inflammatory agent for the treatment of psoriasis; however, skin irritation is one of its side effects. Labrasol^®^ (47.25%), Plurol^®^ isostearique (15.75%), and water (2%) were used as the surfactant, co-surfactant, and aqueous phases, respectively, to create jojoba oil-based w/o ME in which tazarotene (0.1% *w*/*w*) was encapsulated. The potential to cause irritation and the therapeutic outcomes were compared between the commercial anti-psoriasis gel and the ME formulation by evaluating their effects on 20 patients with psoriasis. The findings revealed that patients who used the ME formulation experienced no irritation, whereas those who used the commercial gel treatment experienced redness and inflammation [107]. A similar finding was reported for jojoba oil-based w/o ME containing tazarotene (0.1% *w*/*w*) using Tween^®^ 80 (30%), Span^®^ 85 (15%), and water (15%) as the surfactant, co-surfactant, and aqueous phases, respectively. The ME formulation was then applied on the inner arm skin of human volunteers for assessment of skin irritancy. The results indicated a lack of erythema or erosion symptoms after 24 h of application [108]. Methotrexate (MTX) has been approved by the United States Food and Drug Administration for the treatment of psoriasis. Since the use of systemic MTX is associated with several adverse effects, topical MTX has gained popularity as a potential alternative. MTX-loaded jojoba oil-based w/o ME was developed using Tween^®^ 80 (30%), Span^®^ 85 (15%), and water (15%) as the surfactant, co-surfactant, and aqueous phases, respectively. Thirty patients with plaque psoriasis underwent an 8-week course of treatment to determine whether they experienced skin irritation or allergic contact sensitization. The results indicated that topical ME dramatically reduced the psoriatic plaques without causing skin irritation or adverse effects [109].

## 5. Inflammation and Pathways

**Figure 5:**
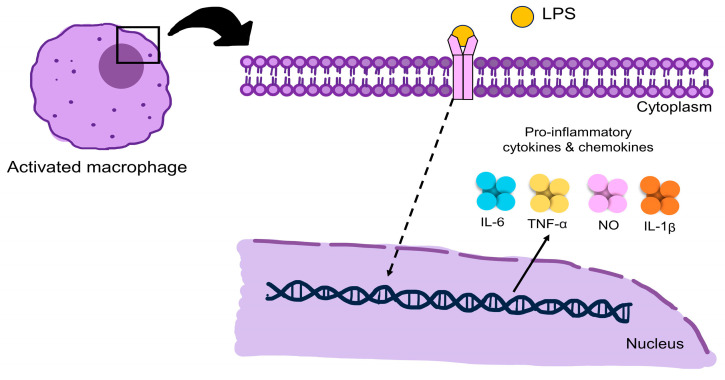
Schematic picture proposes the LPS-stimulated inflammatory response in RAW 264.7 macrophages. LPS: lipopolysaccharide; IL-6: interleukin-6; TNF-α: tumor necrosis factor-alpha; NO: nitric oxide; IL-1β: interleukin-1 beta.

Inflammation is a natural immune response that protects the body against pathogens, damage, and toxic substances. Its purpose is to eliminate threats, promote tissue repair, and prevent harm or infection [110,111]. There are two types of inflammation, acute and chronic. Acute inflammation is a crucial component of innate immunity response, serving as a defense mechanism against invading pathogens and harmful chemicals. The symptoms include redness, discomfort, swelling, pain, and heat. Chronic inflammation can last for months or even years and is characterized by a prolonged response [112,113,114]. Various techniques are employed to assess the ability of substances to induce inflammation, including in vitro assays using RAW 264.7 and THP-1 cells. RAW 264.7 cells are from mice, whereas THP-1 is a human myeloid leukemia mononuclear cell line [115,116]. These cells generate strong inflammatory responses when exposed to stimuli, such as lipopolysaccharide (LPS), leading to the production of various inflammatory mediators, such as nitric oxide (NO), TNF-α, IL-1β, and IL-6 (Figure 5) [117,118].

Additionally, animal models have been widely used to study anti-inflammatory activities. In comparison to in vitro models, animal models offer a more comprehensive understanding of the physiological response to anti-inflammatory treatments. This is because animal models involve complex interactions between various cell types, tissues, and organ systems that cannot be replicated in in vitro studies. Consequently, the use of animal models provides a deeper understanding of the mechanism of the immune system in its response to anti-inflammatory treatments. Several animal models are employed in anti-inflammatory research, including carrageenan-induced paw edema, histamine/serotonin (5-HT)-induced paw edema, bradykinin-induced paw edema, dextran-induced paw edema, LPS-induced paw edema, and arachidonic acid (AA)-induced ear edema, among others [119,120,121].

## 6. Anti-Inflammatory of MEs Containing Herbal Substances

Table 2 shows studies exploring the anti-inflammatory potential of MEs containing herbal substances. For instance, Chittasupho et al. [41] investigated the in vitro anti-inflammatory activity of MEs containing *Kaempferia galanga* rhizome oil. The formulated ME comprised *K. galanga* rhizome oil (27.27%), Tween^®^ 80 (63.64%), and water (9.09%). The ME formulation exhibited enhanced anti-inflammatory activity compared to *K. galanga* rhizome oil alone, as evidenced by a lower concentration required to achieve a 50% reduction in NO secretion (203.0 µg/mL) compared to the pure oil (770.5 µg/mL).

Benjakul (BJK) is a combination of five botanical herbal constituents commonly used in traditional Thai medicine for its analgesic and anti-inflammatory effects. The anti-inflammatory activity of the BJK ethanolic extract was reported in LPS-treated Caco-2 cells by Burodom and Itharat [121]. Kuropakornpong et al. [122] developed topical MEs containing BJK, which exhibited higher anti-inflammatory activity than BJK extract alone. This was evidenced by a reduction in NO production in RAW 264.7 macrophage cells treated with the MEs containing ethanolic BJK extract (BJK-MEs). The optimized BJK-ME contained 5% isopropyl myristate (IPM), 30% Labrasol^®^, 10% Transcutol^®^, and 55% water. Following six months of storage under accelerated conditions, there were no significant alterations in its anti-inflammatory activity compared to the initial time (*p* > 0.05).

Ginger is known for its anti-inflammatory properties due to the presence of gingerols, shagaols, and paradols. However, instability in the presence of light, air, heat, and storage has been observed. Akram et al. [123] incorporated ginger into an ME. The optimized ME formulation contained 20% Tween^®^ 80, 20% PEG 400, 4% IPM, and 56% water. The ginger-loaded ME exhibited superior anti-inflammatory activity compared to the piroxicam solution, inhibiting protein denaturation more effectively at concentrations of 31.25 to 1000 µg/mL (*p* < 0.05). The ginger extract-loaded MEs were stable for 90 days at room temperature.

Reis et al. [124] studied the effectiveness of babassu oil in mice ear edema and created babassu oil-loaded MEs for topical use because of their anti-inflammatory activities. The authors found that babassu oil at doses of 3 and 10 μL/ear resulted in a reduction of phorbol 12-myristate 13-acetate-induced ear edema by 19.1 and 54.1%, respectively. Lauric acid, a key fatty acid in babassu oil, is believed to play a crucial role in its anti-inflammatory properties. Both the application of the oil at a dose of 10 μL/ear and the use of its component, lauric acid—at a dose of 4 mg/ear—exhibited significant inhibition of edema in the AA, ethyl phenylpropiolate, and phenol-induced ear edema models. The incorporation of 12.2% babassu oil into MEs, comprising of Span^®^ 80 (29.28%) and Kolliphor^®^ EL (19.52%), exhibited higher anti-inflammatory activity than babassu oil at the same concentration diluted with acetone (17.5% vs. 66.2%).

A study by Pascoa et al. [125] examined the anti-inflammatory potential of *Pterodon emarginatus* (“sucupira”) oil in a ME formulation comprising 10% sucupira oil and a mixture of ethoxylated castor oil (10% Ultramone^®^ R-540 and 5% PG). Inflammation was induced in the mice using 2.5% croton oil in acetone, which caused ear edema and inflammation. The mice were treated with either sucupira oil or sucupira oil-loaded MEs, and ear edema was measured in both groups. Both the pure oil as well as oil-loaded MEs demonstrated significant anti-inflammatory effects, but the MEs exhibited significantly greater efficacy than pure oil.

Chaiyana et al. [126] analyzed the composition of *Zingiber cassumunar* essential oil (EO) and found that it contained terpinen-4-ol and sabinene. These compounds exhibited anti-inflammatory activities, as demonstrated by the significant reduction in nuclear factor-kappa B (NF-κB) expression in U937 cells accessed via Western blotting. Moreover, they exhibited a reduction in IL-6 levels in LPS-treated RAW 264.7 cells. Due to the instability of EO, it was formulated into an ME to enhance its stability. The EO-loaded ME comprised EO, Tween^®^ 20 and PG (2:1), and water. The authors observed that incorporating EO into MEs provided protection against the evaporation of sabinene, leading to enhanced chemical stability and increased anti-inflammatory activity. This was supported by the inhibition of albumin denaturation and NF-κB expression in LPS-treated RAW 264.7 cells. The higher effectiveness of this ME was attributed to small droplet size and large surface area, which facilitated contact with the cells.

Triastuti et al. [127] developed MEs containing 0.3% *Plantago major* L. extract. The anti-inflammatory activity of the formulated MEs was tested using a croton oil-induced mouse ear edema model and compared with that of hydrocortisone (1 mg). After 4 h of croton oil application, inflammation was measured. MEs loaded with the extract reduced ear edema and the number of pro-inflammatory cells in mice, and the observation was similar to 1% hydrocortisone.

Oliveira Neves et al. [128] also developed two o/w MEs containing *Copaifera multijuga* oil resin (COR) to enhance the stability and to deliver β-caryophyllene (β-CP), a bioactive compound found in COR, which is highly unstable owing to the presence of unsaturated hydrocarbons in its structure. ME-I contained 8.5% Plurol^®^ Oleique 5203, 33.8% Labrasol^®^, and 10.6% COR, whereas ME-II contained 18.3% Plurol^®^ Oleique 5203, 36.6% Labrasol^®^, and 6.1% COR. Anti-inflammatory activity was assessed using the rat paw edema test, which involved the administration of 1% *w/v* λ-carrageenan solution in saline to induce edema. A plethysmometer was used to measure the paw volume at 1.5, 3.0, and 5.0 h after the injection of λ-carrageenan. The results revealed that both the ME formulations exhibited anti-inflammatory effects, with ME-II demonstrating the strongest effect after 3 h (greater than that of the diclofenac diethylamine emulgel and ME-I). The enhanced anti-inflammatory activity of ME-II was attributed to its higher surfactant content, which may have enhanced the β-CP release and absorption. The results of this study demonstrated that MEs could potentially serve as a delivery system for β-CP in traditional medicine.

The topical delivery of ursolic acid (UA), a promising natural pentacyclic triterpenoid carboxylic acid compound that has anti-inflammatory activity, is challenging because of its poor absorption and low bioavailability. Fonseca-Santos et al. [129] formulated o/w MEs to improve UA delivery, and the anti-inflammatory activity of MEs was then evaluated using the croton oil-induced ear edema technique. MEs were created by solubilizing UA in oleic acid using Procetyl^®^ AWS as a surfactant. Two ME formulations (ME-A and ME-B) were prepared using different ratios of oleic acid (ME-A = 40% and ME-B = 30%) and water (ME-A = 10% and ME-B =20%). Both ME-A and ME-B demonstrated anti-inflammatory effects in the mouse model of ear edema. The treatment resulted in approximately 60 and 50% inhibition by ME-A and ME-B, respectively, compared to 25% edema inhibition by dexamethasone. The findings of this study suggested that incorporating UA into MEs has the potential to be an effective treatment option for skin inflammation.

## 7. Future Perspective

MEs have garnered considerable attention as lipid-based drug delivery systems, generating substantial interest among researchers. They have been extensively used for the delivery of drugs both systemically (transdermal) and locally (topical) through the skin. Furthermore, encapsulating herbal substances in MEs provides a means to improve stability and achieve prolonged release. In the context of topical applications, MEs have the potential to enhance skin penetration, leading to increased efficacy of the encapsulated herbal substances. Despite the high concentration of nonionic surfactants in MEs, several studies have confirmed their safety for topical use, as described earlier. The compelling evidence from these studies strongly supports and advocates the use of MEs for topical application. Additionally, the absence of skin irritation has been observed in the long-term application of MEs (for a period of 12 weeks) [100]. The ingredients used in ME formulations are generally approved for use in cosmetics and topical pharmaceuticals. Additionally, MEs are advantageous in terms of production as they can be easily produced using simple agitation methods and consume less energy. Consequently, there is substantial evidence suggesting that MEs have the potential to serve as efficient topical drug delivery systems in the future. This is particularly relevant for herbal cosmeceuticals, as the MEs are anticipated to enhance the delivery of active herbal substances through the stratum corneum of the skin, thereby improving their therapeutic efficacy compared to conventional formulations.

## 8. Conclusions

MEs are colloidal drug carrier systems that are transparent, thermodynamically stable, and widely used by scientists for efficient drug administration across the skin. They are isotropic mixtures of hydrophilic and lipophilic molecules stabilized by suitable surfactants and/or co-surfactants. Compared to conventional carrier systems, MEs offer advantages such as ease of manufacture, long-term stability, improved solubilization, biocompatibility, skin-friendly appearance, and compatibility with both hydrophilic and lipophilic active compounds. Notably, the topical administration of many herbal substances is limited because of the skin’s permeability barrier, particularly in the stratum corneum. To overcome this limitation, researchers have turned to MEs as practical and cost-effective carrier systems for delivering herbal substances to and through the skin. The enhanced skin penetration of herbal substance-loaded MEs can be attributed to the presence of high amounts of surfactants, which can effectively disrupt the skin barrier. Extensive research has been conducted to assess the potential for skin irritation associated with herbal substance-loaded MEs. The findings indicate that MEs loaded with herbal substances do not cause or increase skin irritation compared to the herbal substances alone. In fact, some studies have reported that encapsulating herbal substances in MEs can reduce skin irritation caused by the ingredients themselves. Furthermore, MEs have been reported to enhance the efficacy of herbal substances, particularly in terms of their anti-inflammatory activity when used as a delivery system. In summary, MEs offer a promising avenue as colloidal carriers for delivering herbal substances.

## Figures and Tables

**Table 1:** Examples of the skin irritation potential of topical MEs containing herbal substances.

Topical MEs	Indications	ME Components	Physicochemical Properties	Main Results	References
*Moringa oleifera* seed oil-loaded ME	Anti-inflammatory activity	*M. oleifera* seed oil, Tween^®^ 80, Span^®^ 80, water	Size: 167 nm PDI: 0.30 ZP: –35.8 mV pH: 7.1 RI: 1.469 η: 88.7 mPa.s	- Non-irritating effect on rabbit skin - ↑ In vitro drug release through a cellulose acetate membrane - ↑ Anti-inflammatory effect determined by evaluating vascular permeability and carrageenan-induced paw edema in rats	[97]
Salvianolic acid B-loaded ME	Anti-psoriatic effects	Silicon oil AR200, squalene, triglyceride, PEG-40 castor oil, Tween^®^ 80, PEG 400, 1,2-propylene glycol, sorbitol, glycerol, phosphate-buffered saline	σ: 24 μS/cm Size: 696 nm PDI: 0.44 ZP: –15.0 mV η: 3112.3 mPa.s	- ↓ Skin irritancy in an imiquimod-induced psoriasis-like mouse skin - ↑ In vitro skin penetration and deposition through mouse skin - ↑ Anti-inflammatory effect in mouse skin by downregulating the IL-23/IL-17 axis and inhibiting the abnormal proliferation of keratinocytes - ↑ Skin hydration in mouse skin	[98]
*Cordyceps militaris* extract-loaded ME serum	Anti-skin wrinkle properties	Sugar squalane, Tween^®^ 85, propylene glycol, water	Size: 146 nm PDI: 0.50	- Non-irritating effect on human volunteers - ↑ Skin moisturization and elasticity in human volunteers	[99]
*Punica granatum* extract-loaded ME	Control of skin erythema and melanin	Palm oil, Tween^®^ 80, propylene glycol, water	σ: 6 μS/cm Size: 8 nm PDI: 0.36 pH: 5.7	- Non-irritating effect on human volunteers - ↓ Skin erythema and melanin in human volunteers	[100]
Catechin-loaded ME	Not specified	Isopropyl myristate, Brij 35, Brij 30, ethanol, water	Size: 369 nm η: 9.3 mPa.s	- Non-irritating effect on rat skin - ↑ In vitro skin penetration and deposition, and ↓ lag time in rat skin - Physicochemically stable after 3-month storage at 25 ± 2 °C/60 ± 5% RH	[101]
Dibenzoylmethane-loaded ME	Treatment of UV-induced photoaging	Captex 300, Tween^®^ 80, n-butanol, menthol, water	Size: 36 nm PDI: 0.28 ZP: 0.4 mV pH: 6.8 η: 45.9 mPa.s	- Non-irritating effect on mouse skin - ↑ Ex vivo skin permeation and deposition through mouse skin - ↑ In vivo anti-photoaging effect on mouse skin using visual scoring, pinch test, biochemical estimations, and histopathological studies - Physicochemically stable after 3-month storage at 4, 25 and 40 °C	[102]
Clove oil-loaded ME gel	Treatment of superficial fungal infections	Clove oil, Tween^®^ 80, isopropyl alcohol, water	σ: 143 μS/cm Size: 14 nm PDI: 0.01 ZP: –0.7 mV pH: 6.0	- Non-irritating effect on rat skin - ↑ In vitro drug release through a cellulose acetate membrane - ↑ Ex vivo skin permeation and deposition in rat skin - ↑ In vitro anti-*Candida* activity examined using agar well method - Physicochemically stable after 12-month storage at 25 ± 2 °C/60 ± 5% RH, 30 ± 2 °C/65 ± 5% and 40 ± 2 °C/75 ± 5% RH	[103]
*Pouteria macrophylla* fruit extract-loaded ME	Treatment of cutaneous depigmentation	Ethyl oleate, Cremophor^®^ ELP, Span^®^ 80, HEPES buffer (pH 4.5)	Size: 49 nm PDI: 0.35 ZP: –25.0 mV pH: 4.0	- Non-irritating effect on RhE (EpiSkin™) model - Physiochemically stable after 1-month storage at room temperature (20–25 °C) - ↓ In vitro drug release through a hydrophilic cellulose membrane (controlled release system) - ↑ In vitro skin deposition through pig skin - ↑ Depigmenting effect in 3D pigmented RhE model	[104]

ME: microemulsion; PEG: polyethylene glycol; HEPES: 2-[4-(2-hydroxyethyl)-piperazin-1-yl]-ethanesulfonic acid; σ: conductivity; PDI: polydispersity index; ZP: zeta potential; RI: refractive index; η: viscosity; IL: interleukin; RhE: reconstructed human epidermis; RH: relative humidity.

**Table 2:** Examples of the anti-inflammatory potential of topical MEs containing herbal substances.

Topical MEs	Anti-Inflammatory Substances	ME Components	Physicochemical Properties	Main Results	References
*Kaempferia galanga* oil-loaded ME	*K. galanga* rhizome oil	*K. galanga* oil, Tween^®^ 80, water	σ: 54,400 μS/cm Size: 216 nm PDI: 0.40 ZP: –13.8 mV pH: 6.7	- ↑ Anti-inflammatory effect evidenced by inhibition of nitric oxide production against lipopolysaccharide-stimulated RAW 264.7 cells - Moderate sun protection (in vitro)	[41]
Benjakul-loaded ME	Benjakul: A combination of five botanical herbal constituents (*Piper chaba*, *Piper sarmentosum*, *Piper interuptum*, *Plumbago indica*, and *Zingiber officinale*)	Isopropyl myristate, Labrasol^®^, Transcutol^®^, water	σ: 46 μS/cm Size: 279 nm PDI: 0.39 pH: 3.8	- ↑ Anti-inflammatory effect evidenced by inhibition of nitric oxide production against lipopolysaccharide-stimulated RAW 264.7 cells - No in vitro skin cell toxicity was observed in human adult keratinocytes (HaCaT) - Chemically and biologically stable after 6-month storage at 40 ± 2 °C/75 ± 5% RH	[122]
Ginger-loaded ME	*Zingiber officinale* rhizome extract	Isopropyl myristate, Tween^®^ 80, polyethylene glycol 400, water	σ: 207 μS/cm Size: 22 nm PDI: 0.16 ZP: –22.8 mV pH: 5.9 RI: 1.397 η: 26.0 mPa.s	- ↑ In vitro anti-inflammatory effect demonstrated by protein denaturation studies - Physicochemically stable after 3-month storage at room temperature (25.0 ± 0.5 °C)	[123]
Babassu oil-loaded ME	Babassu oil	Babassu oil, Span^®^ 80, Kolliphor^®^ EL, propylene glycol, water	σ: 21 μS/cm Size: 5–15 nm η: 300.0 mPa.s	- ↑ Anti-inflammatory activity evidenced by inhibition of phorbol 12-myristate 13-acetate-induced ear edema in mice	[124]
*Pterodon emarginatus* oil-loaded ME	*P. emarginatus* oil	*P. emarginatus* oil, Ultramone R-540^®^, propylene glycol, water	Size: 57 nm PDI: <0.20 ZP: –11.3 mV pH: 5.7	- ↑ Anti-inflammatory activity evidenced by inhibition of croton oil-induced ear edema in mice - Physically stable after 1-month storage at 5 ± 2 °C and 25 ± 2 °C	[125]
*Zingiber cassumunar* oil-loaded ME	*Z. cassumunar* rhizome oil	*Z. cassumunar* oil, Tween^®^ 20, propylene glycol, water	Size: 212–367 nm PDI: <0.38 η: 0.7–0.8 mPa.s	- ↑ Anti-inflammatory activity evidenced by the evaluation of nuclear factor-kappa B levels in human leukemic monocyte lymphoma (U937) cells by Western blot analysis and albumin denaturation inhibition analysis - Physicochemically stable after six heating–cooling cycles consisting of 24 h each at 4 and 45 °C - No in vitro cell toxicity was observed in human peripheral blood mononuclear cells	[126]
*Plantago major* extract-loaded ME	*P. major* extract	Isopropyl myristate, Span^®^ 80, Tween^®^ 80, propylene glycol, propylparaben, methylparaben, water	Size: 54 nm PDI: 0.40 pH: 6.9 η: 122.8 mPa.s	- ↑ Anti-inflammatory activity evidenced by the inhibition of croton oil-induced ear edema in mice - Physically stable after 4-week storage at 4, 25 and 40 °C	[127]
*Copaifera multijuga* oil-resin-loaded ME	*C. multijuga* (copaiba) oil-resin	Copaiba oil-resin, Plurol^®^ Oleique 5203, Labrasol^®^, water	σ: 121 μS/cm (ME-I), 79 μS/cm (ME-II) Size: 165 nm (ME-I), 199 nm (ME-II) ZP: –2.6 mV (ME-I), –3.6 mV (ME-II) pH: 5.3 (ME-I), 5.7 (ME-II) RI: 1.412 (ME-I), 1.426 (ME-II) η: 64.4 mPa.s (ME-I), 118.8 mPa.s (ME-II)	- ↑ Anti-inflammatory activity evidenced by the inhibition of carrageenan-induced paw edema in rats - ↑ In vitro antimicrobial activity against *Staphylococcus aureus*, *Escherichia coli*, *Pseudomonas aeruginosa*, and *Cryptococcus neoformans* examined using broth dilution method	[128]
Ursolic acid-loaded ME	Ursolic acid	Oleic acid, Procetyl^®^ AWS, water	Yield shear stress: 1.0245 Pa (ME-A), 0.0000 Pa (ME-B) Consistency index: 0.0890 Pa.s^n^ (ME-A), 0.3777 Pa.s^n^ (ME-B) Flow index: 1.0628 (ME-A), 0.9077 (ME-B) S constant (relating to gel strength): 0.0023 (ME-A), 1182.0945 (ME-B) Viscoelastic exponent: 2.4691 (ME-A), 0.2531 (ME-B)	- ↑ Anti-inflammatory activity evidenced by the inhibition of croton oil-induced ear edema in mice	[129]

ME: microemulsion; σ: conductivity; PDI: polydispersity index; ZP: zeta potential; RI: refractive index; η: viscosity; RH: relative humidity.

## Data Availability

Not applicable.
